# Finite Element Analysis of Subperiosteal Implants in Edentulism—On the Basis of the MaI Implant^®^ by Integra Implants^®^

**DOI:** 10.3390/ma16237466

**Published:** 2023-11-30

**Authors:** Rafal Zielinski, Jerzy Sowinski, Martyna Piechaczek, Jakub Okulski, Marcin Kozakiewicz

**Affiliations:** 1 Stomatologia na Ksiezym Mlynie, 16D Tymienieckiego, 90-365 Lodz, Poland; martyna.piechaczek2001@gmail.com; 2 Department of Maxillofacial Surgery, Medical University of Lodz, 113st Zeromskiego, 90-001 Lodz, Poland; jakub.okulski@gmail.com (J.O.); marcin.kozakiewicz@umed.lodz.pl (M.K.); 3 Private Dental Clinic, Tetmajera 3A Rd, 05-080 Izabelin C, Poland; jersow@gmail.com

**Keywords:** subperiosteal implant, custom implant, Mai Implants^®^, Integra Implants^®^, individual implant, FEA of dental implants

## Abstract

The MaI Implants^®^ method offers a modern treatment option for specific patients who lack sufficient bone for traditional screw-based implants. The aim of the article is to use Finite Element Analysis (FEA) to examine the behavior of a subperiosteal implant under actual conditions within the oral cavity and to assess the impact of various mechanical factors on the durability of the MaI Implants^®^. A strength analysis was conducted using Finite Element Analysis for two models. The first was a single subperiosteal implant, while the second was a model of an arch consisting of two single subperiosteal implants connected by a bar. Based on the obtained results, it can be observed that the increase in load from 100 N to 800 N leads to an increase in displacements throughout the implant. Changing the angle from 90 to 30 degrees resulted in a 576% increase in the average displacement value across all multi-units. Stresses in the multi-units range from 23.7 MPa to 268.5 MPa. The lack of proper stabilization of the implant has the greatest impact on the results of displacements. Such displacements are significant for the later positioning of the implant compared to the initial conditions.

## 1. Introduction

Patients with Cawood and Howell classes from V to VI have limited options of using permanent prosthodontic reconstruction on dental implants. Currently, the gold standard of bone reconstruction is still autogenous bone. Donor sites are, in most cases, the anterior iliac crest or calvarial bone grafts [1]. The main disadvantages of autografts include the need for a second surgical site and the morbidity related to bone harvesting [2].

Moreover, elderly patients are poor candidates for bone augmentation due to their decreased metabolic rate and decreased regenerative capacity [3]. When bone augmentation is not feasible, there are alternatives such as subperiosteal or zygomatic implants.

Another way to help edentulous patients with an atrophied maxilla is to apply zygomatic implants; however, these have a high risk of different complications such as prosthesis failure, zygomatic implant fracture, paresthesia/dysesthesia, oro-antral communication, sinusitis, infection located at the apex of an implant, peri-implant mucositis, peri-implantitis, and retraction of buccal/labial peri-implant tissue or penetration into the orbital cavity [4,5].

The clinical use of subperiosteal implants was largely abandoned over time with the advent of endosseous titanium implants. However, it is not possible to place these in patients with Cawood and Howell classes V–VI unless extensive bone grafting is performed.

Endosseous implants often integrate successfully with bone, but their placement and lasting success rely on having adequate bone quality and volume. Situations like significant bone loss due to disuse, trauma, or tumors can make it challenging or even unfeasible to place these implants [5,6,7]. Even after placing screw-type implants, whether in original alveolar bone or bone regenerated after grafting, the alveolar bone can continue to deteriorate [8].

Thanks to advancements in digital technology, we can now create custom-made implants for individual patients. The breakthroughs in 3D printing have also revived older ideas, like subperiosteal implants. This has led to the development of the Maxilla/Mandible Personalized Implants (MaI Implants^®^). MaI Implants^®^ are made up of two segments (left and right), tailored to each patient’s skeletal structure using the provided CBCT data. Each segment comprises two wings, a foundational looped frame linking the wings, and the transmucosal multi-units. The wings are positioned at the canine and zygomatic buttress and are anchored there with osteosynthesis screws with a diameter of 2.0 and rescue screws with a diameter of 2.2 in case of the stability being too low. Both segments are initially joined inside the mouth with a provisional acrylic bridge and later replaced by either a metal–porcelain (PFM) bridge, a titanium alloy–acrylic bridge (Ti-acryl), or a titanium–zirconia dioxide–porcelain (Ti-ZrO-porc.) bridge.

**Figure 1:**
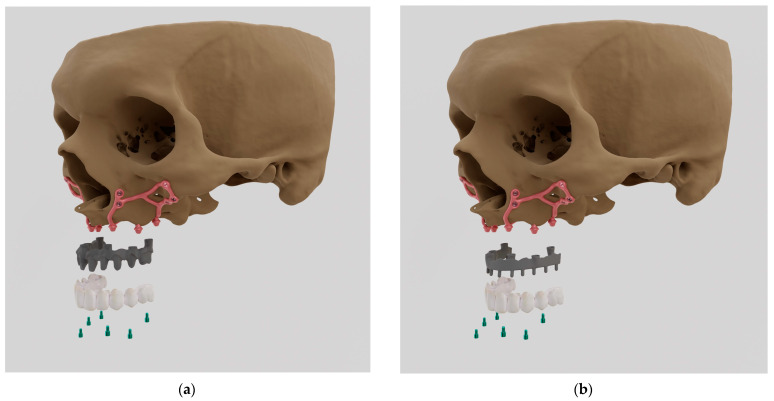
Mai Implants^®^ showing (**a**) milled metal CoCr alloy + porcelain bridge (PFM); (**b**) milled titanium alloy + acryl (Ti-acryl).

The MaI Implants^®^ method offers a modern treatment option for specific patients who lack sufficient bone for traditional screw-based implants (Figure 1). This customized approach not only provides anchorage to the bone but also gives patients immediate functional recovery on the surgery day. Further clinical studies are needed to validate the effectiveness of this updated approach. In the literature, some authors describe the FEA of subperiosteal implants; however, it was not conducted for individual cases and it was designed only for the premolar area [9].

The aim of the article is to use Finite Element Analysis (FEA) to examine the behavior of a subperiosteal implant under actual conditions within the oral cavity and to assess the impact of various mechanical factors on the durability of the MaI Implants^®^. The null hypothesis is that MaI Implants^®^ are not suitable for edentulous reconstruction in the cases of high maxilla atrophy.

## 2. Materials and Methods

### 2.1. Methods

As part of the research section, a strength analysis was conducted using Finite Element Analysis for two models. The first was a single subperiosteal implant, while the second was a model of an arch consisting of two single subperiosteal implants connected by a bar, which was created in the Autodesk Inventor program. Using the Ansys Workbench software 19.4 (Canonsburg, PA, USA), the effect of loading the implants with selected forces and at specific angles on the state of displacements, deformations, and stresses resulting from the simulation was examined. Depending on the model adopted for the study, the simulation consisted of three or four stages.

**Figure 2:**
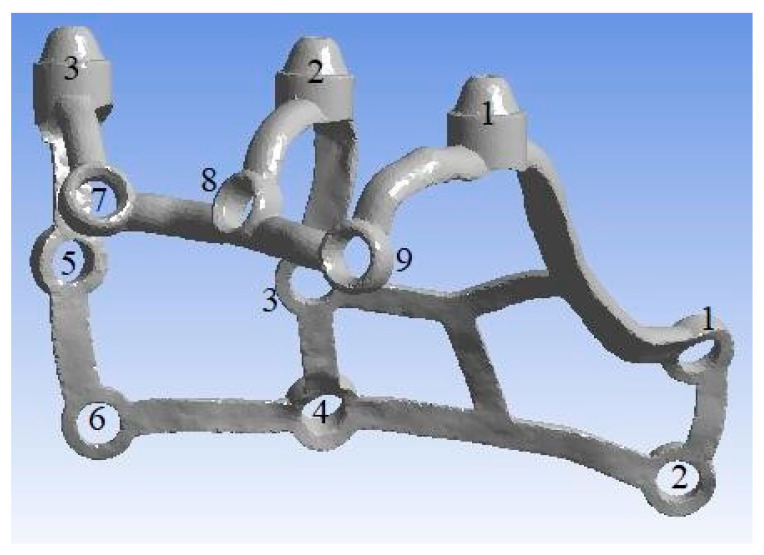
The model of a single subperiosteal implant along with numbering. Anchoring points are numbered from 1–9 as “O-shaped” whereas multi-units are numbered from 1–3 at the top of the figure.

For the model representing the arch, the first step was to create its geometry necessary for performing calculations. Then, both models underwent discretization, which involved generating a computational grid consisting of triangles of optimal sizes to obtain accurate results. The next step was to define boundary conditions, i.e., constant and unchanging restrictions in the appropriate places, intended to reflect real conditions. The final stage was to perform calculations for both models, taking into account the applied grid, as well as the boundary conditions, and a final analysis of the obtained results along with their graphical presentation (Figure 2).

### 2.2. Models

**Figure 3:**
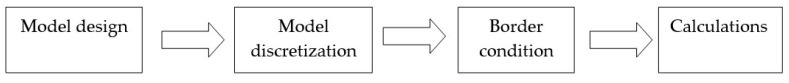
Flow chart showing the process of Finite Element Analysis (FEA).

**Figure 4:**
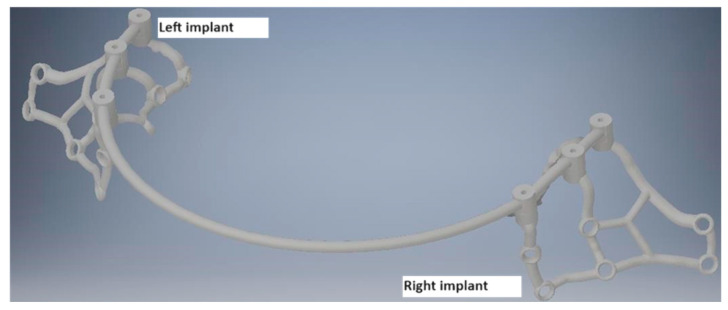
The designed arch along with the division into left and right implants.

To conduct the strength analysis, two models were used: a single subperiosteal implant (Figure 3) and an arch consisting of two implants of the same type connected by a bar (Figure 4). For the subsequent analysis of results, the numbering of multi-units 1–3 and mounting holes 1–9 was additionally carried out for both models, and the arch was detailed into a left implant and a right implant. The geometry of the whole MaI Implant^®^ (Integra Implants, Lodz, Poland) as designed on the basis of the experience of the practitioner, an implantologist and the author of the article. Using Autodesk Inventor software, the arch was designed using the primary subperiosteal implant, upon which a beam was created that was attached to each of the three multi-units, forming an arch connecting both subperiosteal implants. The width of the created arch, measured between the centers of the extreme multi-units of both implants, is about 104 mm, which corresponds to the average anatomical width of the mandible.

### 2.3. FEA Analysis

To conduct the strength analysis, Ansys software was used, which employs Finite Element Analysis (FEA). This method involves replicating the geometry of a given model by generating a computational grid, defining boundary conditions, i.e., loading the structure and securing the model, and also specifying material parameters based on which the simulation is carried out. The object undergoes discretization and is divided into many simple parts (finite elements) of a specific shape and density, determining the accuracy of the obtained results. The denser the grid, the more accurate the results, but this also extends the computation time. The displacements, deformations, and von Mises stresses resulting from the simulation were selected for analysis, indicating the new positions of the grid nodes after the model’s state change.

#### 2.3.1. Model Import and Materials

Importing models into the Ansys Workbench was carried out by creating a “Geometry” block and loading a file in the .step format, then linking it with “Geometry” in the newly created “StaticStructural” block. In “Engineering Data”, a material was assigned, specifically titanium alloy grade 5—Ti-6Al-4V with the following predefined properties according to norm ISO 5832-2:

- Density—4.405 [g/cm^3^],
- Young’s modulus—107 [GPa],
- Yield strength—1092 [MPa],
- Poisson’s ratio—0.323.

#### 2.3.2. Discretization of Models

The selection of the optimal computational grid, which determines the length and accuracy of the calculations, was based on a test analysis for three chosen grid element sizes: 0.2 mm, 0.5 mm, and 1 mm. The first element size resulted in an overly long computation time, while the results obtained between the 0.5 mm and 1 mm grids were similar. Therefore, for a shorter analysis duration, a grid with an element size of 1 mm was chosen. The comparison between the 0.5 mm and 1 mm meshes showed negligible differences in the results. This similarity in outcomes suggests that the 1 mm mesh size is sufficiently fine to capture the necessary detail of the implant geometry without significantly compromising the accuracy of the results. Based on these findings, we chose a 1 mm mesh size for its balance between computational efficiency and accuracy. This decision was not solely based on reducing computation time but also on the observed convergence of results between the 0.5 mm and 1 mm mesh sizes. As a result, the geometry of a single implant was obtained with 128,626 nodes and 71,121 elements, and the full arch was obtained with 282,424 nodes and 156,555 elements.

#### 2.3.3. Border Conditions

**Figure 5:**
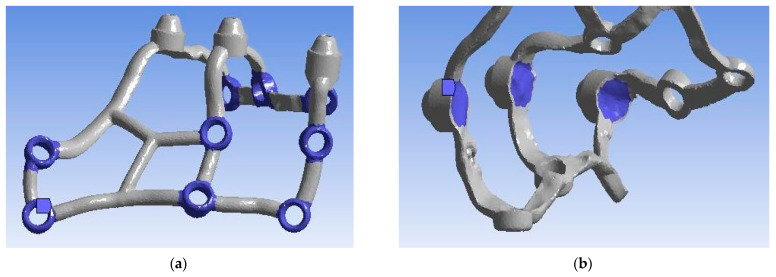
(**a**) Anchoring holes; (**b**) surface adjustment for multi-units.

The formulation of boundary conditions is essential to accurately replicate the conditions prevailing in the oral cavity during the use of a subperiosteal implant. They allow for certain assumptions and simplifications to be made. For both the single implant model and the arch, the holes (Figure 5a) through which screws are introduced to fix the implant to the bone tissue and the surfaces under the multi-units that rest on the bone tissue(Figure 5b) were taken as the anchoring points. The reason for this was osteosynthesis between the bone and the titanium alloy that lies firmly on the alveolar crest of the bone. Moreover, the multi-unit bar is screwed so that multi-units are more unmovable than any other part of the implant.

**Figure 6:**
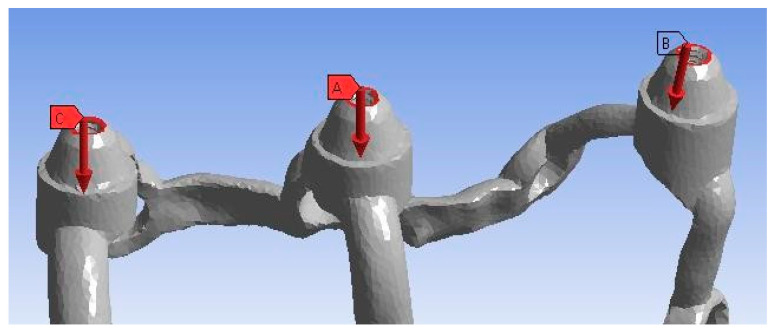
Place and direction of vertical loads for a single model. A, B, C are numbered multi-units.

**Figure 7:**
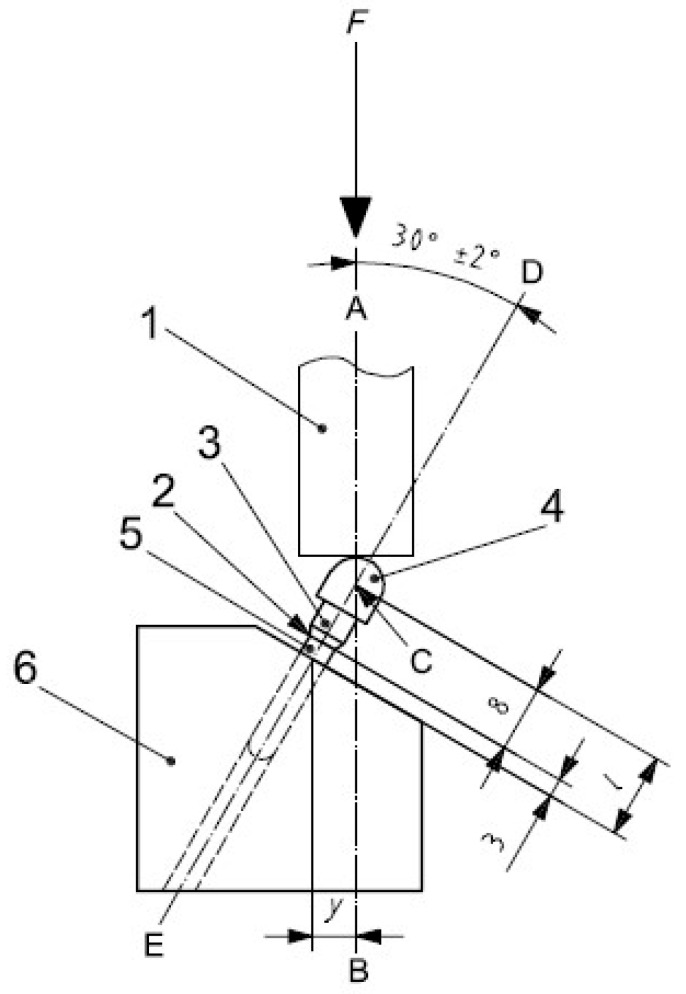
The loading scheme of the implant according to ISO norm 14801.

The load on the implants can be axial or non-axial. An axially applied force is directed along the long axis of the multi-unit, while a non-axial or horizontal load transfers tensile stresses, causing a bending motion, which is considered destructive [10].The site of the load application on a single implant was chosen as the upper surface in all three multi-units (Figure 6), whereas, in the arch, the site was the surfaces on the bar connecting both implants at the height of each of the six multi-units. Based on literature data (Table 1) [11,12,13,14,15,16,17,18] and despite the fact that the load model according to the PN-EN ISO 14801 standard is made for endosseous implants (Figure 7), the following force variants were chosen.

The reason why the PN-EN ISO 14801 standard was selected is because of the lack of any ISO norms or even data in the literature on how to perform virtual fatigue tests for subperiosteal individual maxillofacial implants.

- Loads with values of 100 N and 800 N at an angle of 90 degrees relative to the vertical axis of the multi-units.

#### 2.3.4. Localization of Measurements

**Figure 8:**
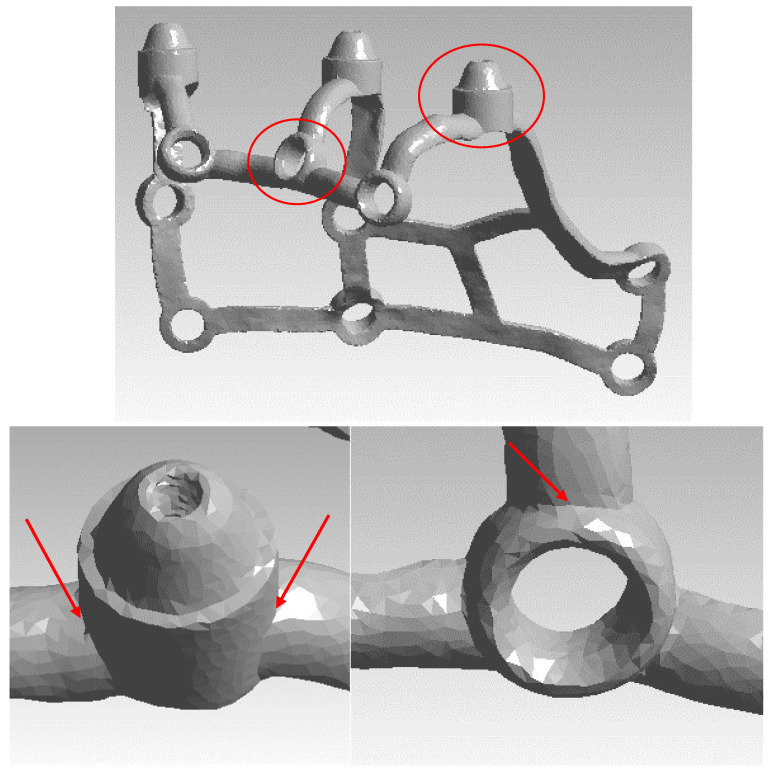
Measurement location in the multi-units and the mounting hole marked by means of red arrows. Two graphs below illustrate in magnification above graph.

**Figure 9:**
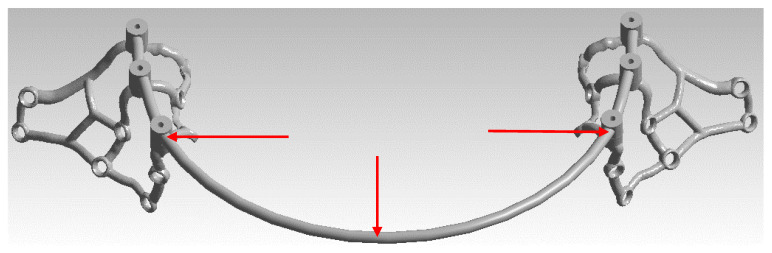
Measurement location in the bar marked by means of red arrows.

The displacements, deformations, and stresses resulting from the application of loads were measured on both the external and internal sides of the single implant multi-units and arch, in the mounting holes, which were characterized by the highest values (Figure 8), and also in selected locations on the beam in the case of the arch model (Figure 9). The external side in both examined models is the side with 6 mounting holes, while the internal side has 3 holes.

## 3. Results

The obtained results of displacements, deformations, and stresses were compiled in tabular form for a single implant and arch. A description of the results for each model was made, and their mutual comparison was presented graphically using charts, as well as drawings from the Ansys program.

### 3.1. Displacement

In Table 2, the displacement results of individual elements of a single implant (multi-unit, mounting holes) and the arch (additionally, the connecting bar) are presented for variable loads of 100 N and 800 N at a 90-degree angle.

In Table 3, the displacement results of selected elements from the studied models (multi-units, mounting holes, and beam) are presented for a load of 800 N at varying angles of 90, 60, and 30 degrees.

**Figure 10:**
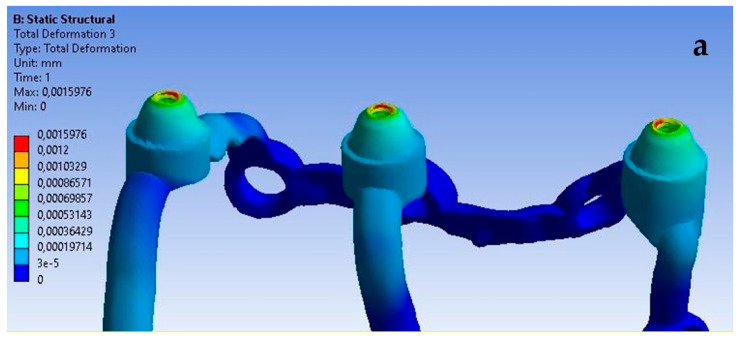

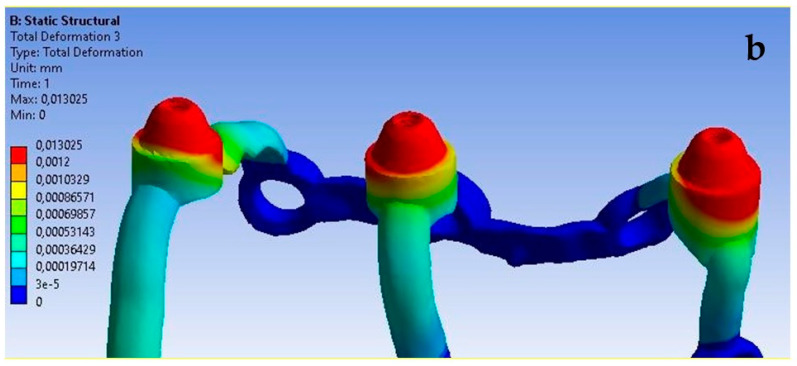
Displacement state for a single implant: (**a**) 100 N, (**b**) 800 N.

Based on the obtained results, it can be observed that the increase in load from 100 N to 800 N leads to an increase in displacements throughout the implant (Figure 10).

**Figure 11:**
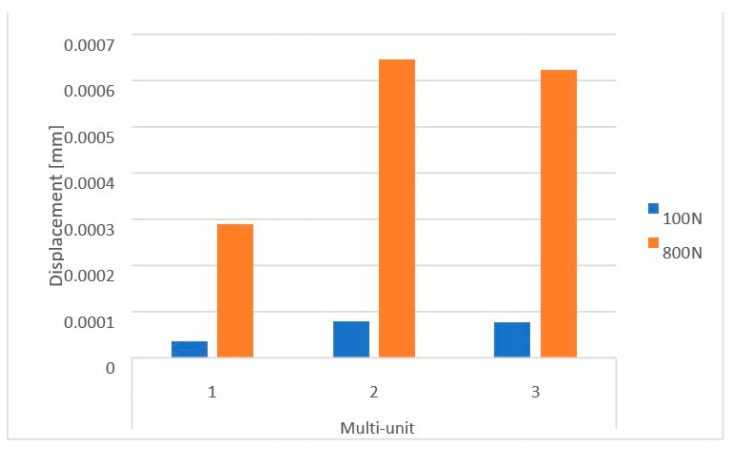
Displacements on the external side of the multi-units of a single implant.

On the external (cheek side) side of the multi-units, loading the implant with two force variants resulted in displacement values ranging from 3.5 × 10^−5^ mm to 6.5 × 10^−4^ mm. The highest displacements occur in the second multi-unit for both the 100 N and 800 N loads (Figure 11). The increase in force caused the average displacement value across all multi-units to rise by 713%.

**Figure 12:**
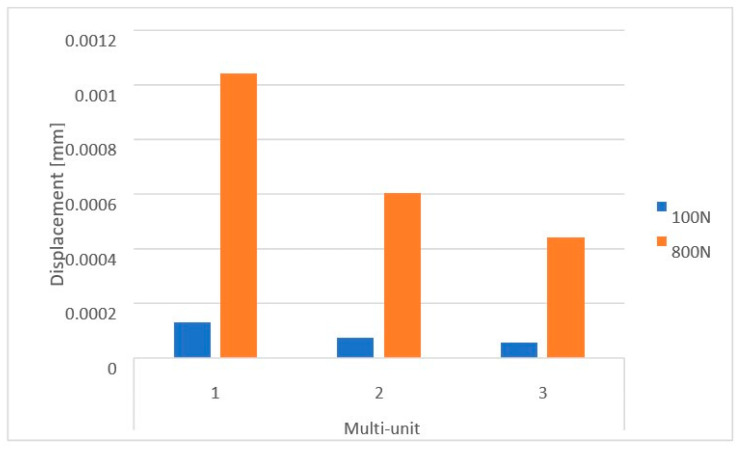
Displacements on the internal side of the multi-units of a single implant.

On the internal (lingual) side, displacement values vary in the range from 5.5 × 10^−5^ mm to 1 × 10^−3^ mm. This range is larger than that observed on the external side. The greatest displacements are seen in the first multi-unit for both force variants (Figure 12). Increasing the load to 800 N caused the average displacement value across all multi-units to rise by 702%. Also located on the internal side is the mounting hole with the highest recorded values, which increased from 2.8 × 10^−6^ to 2.4 × 10^−5^.

**Figure 13:**
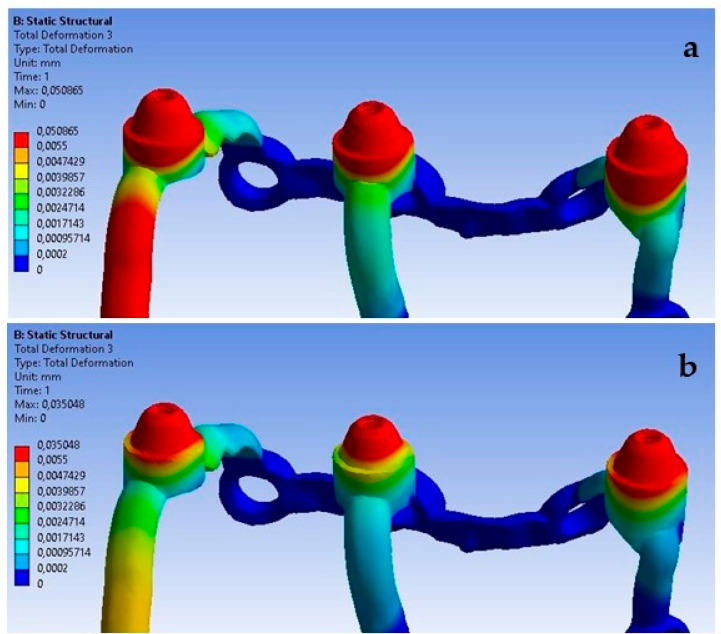

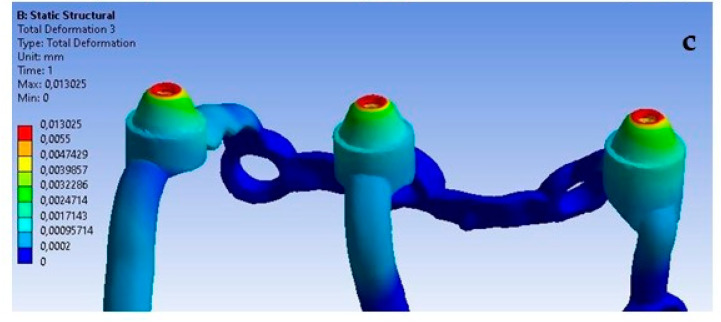
Displacement state for a single implant under an 800 N force at angles of (**a**) 30 degrees, (**b**) 60 degrees, and (**c**) 90 degrees.

Changing the angle between 90 and 30 degrees with a load of 800 N affects the displacement state throughout the single implant. The smallest values are obtained for the load variant at a 90-degree angle and increase as the angle decreases to 60 and then to 30 degrees (Figure 13). The recorded results in the multi-units on both sides range from 2.9 × 10^−4^ mm to 5 × 10^−3^ mm.

**Figure 14:**
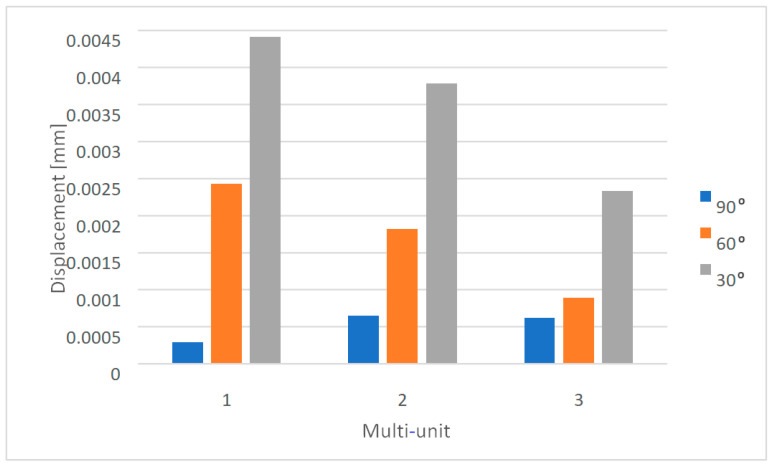
Displacements on the external side of the multi-units for a single implant.

On the external side of the implant, the largest displacements occur in the first multi-unit for angle variants of 60 and 30 degrees. In the case of a 90-degree angle, the highest values are seen in the second multi-unit (Figure 14). Changing the angle from 90 to 30 degrees resulted in a 576% increase in the average displacement value across all multi-units.

**Figure 15:**
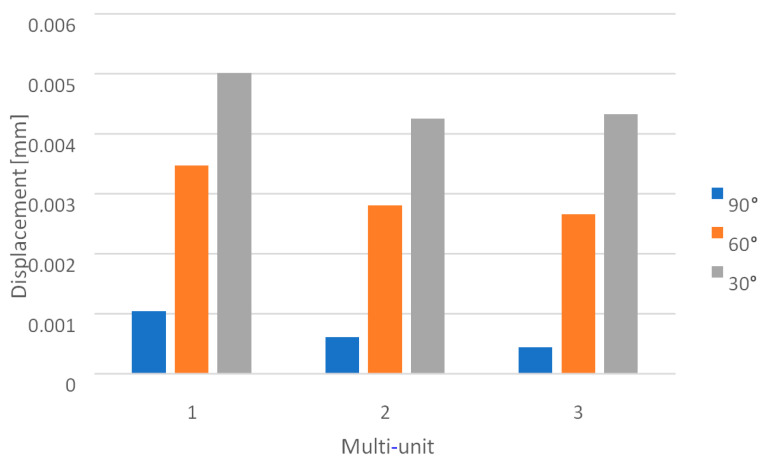
Displacements on the internal side of the multi-units for a single implant.

On the internal side, the largest displacements are observed in the first multi-unit for every angle variant (Figure 15). Changing the angle from 90 to 30 degrees resulted in a 551% increase in the average displacement value across all multi-units. The results in the mounting hole increased from 2.4 × 10^−5^ mm to 1.7 × 10^−4^ mm.

Loading the arch with an increasing force from 100 N to 800 N at a 90-degree angle leads to an increase in displacements throughout the model (Figure 12). On both sides of the multi-units, results vary in the range from 2.1 × 10^−5^ mm to 7.9 × 10^−4^ mm, on the beam from 6.8 × 10^−4^ mm to 1.8 × 10^−2^ mm, while in the selected mounting holes, they range from 2.5 × 10^−6^ mm to 4.4 ×10^−5^ mm.

In both the left and right implants of the arch, the third multi-unit on the external side is characterized by the highest displacement values for each load (Figure 13), while on the internal side, it is the first multi-unit (Figure 14). The highest values also occur in the eighth hole for both implants, which is located on the internal side of the second multi-unit.

Similarly, the change in angle between 90 and 30 degrees with a load of 800 N impacts the results obtained across the entire arch (Figure 15). On both sides of the multi-units, values vary in the range from 1.6 × 10^−4^ mm to 6 × 10^−3^ mm, on the beam from 5.4 × 10^−3^ mm to 1.6 × 10^−1^ mm, and in the mounting holes, they range from 1.4 × 10^−5^ mm to 4.6 × 10^−4^ mm. For each element, the results are greater compared to the variable load at a 90-degree angle.

**Figure 16:**
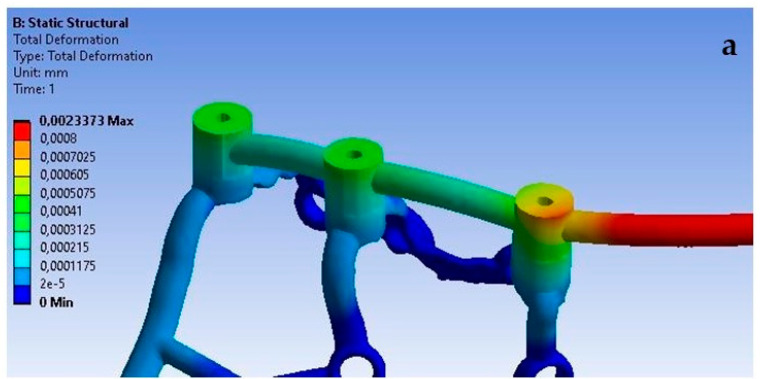

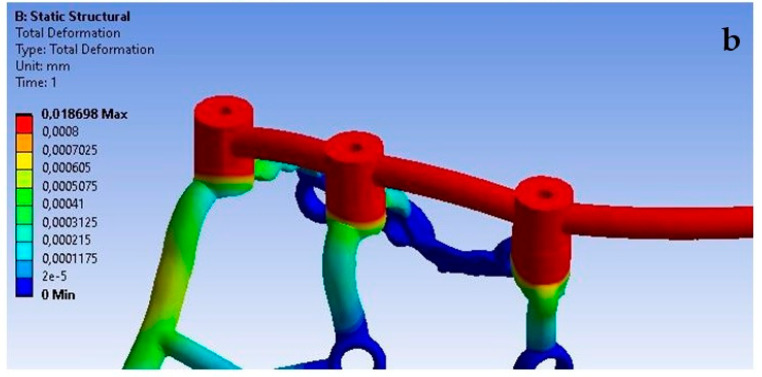
Displacement state for the arch under load: (**a**) 100 N, (**b**) 800 N.

**Figure 17:**
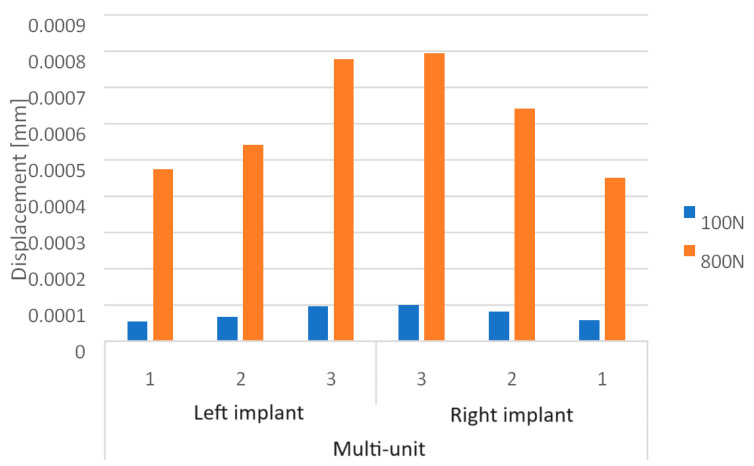
Displacements on the external side of the multi-units for the arch.

On the external side, for the angle variants of 60 and 30 degrees, the most significant displacements in the left implant occur in the first multi-unit, while in the right implant, they are seen in the second multi-unit. For the 90-degree angle variant, it was the third multi-unit in both implants (Figure 16). On the internal side, the largest values for the angles of 60 and 30 degrees were observed in the second multi-units, whereas for the 90-degree angle, they were in the first ones (Figure 17). An increase in displacements was also noted on the connecting beam of the implants. Directly adjacent to the third multi-unit of each implant, they increased from 5.5 × 10^−3^ mm to 4 × 10^−2^ mm, while in the middle of the beam they went from 1.8 × 10^−2^ mm to 0.2 mm. The results are more significant compared to the variable load value at a 90-degree angle.

**Figure 18:**
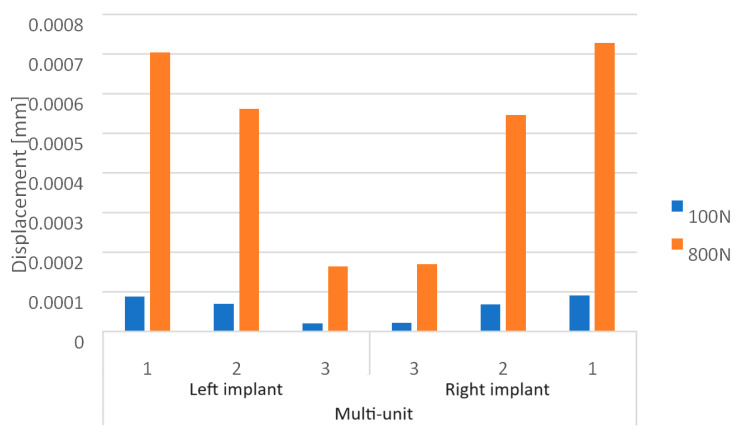
Displacements on the internal side of the multi-units for the arch.

**Figure 19:**
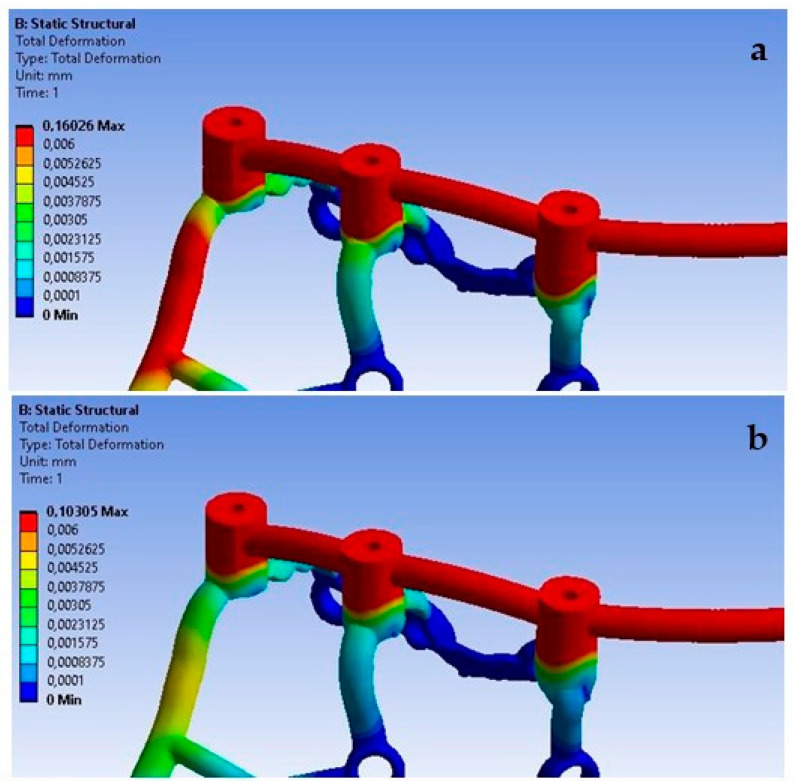

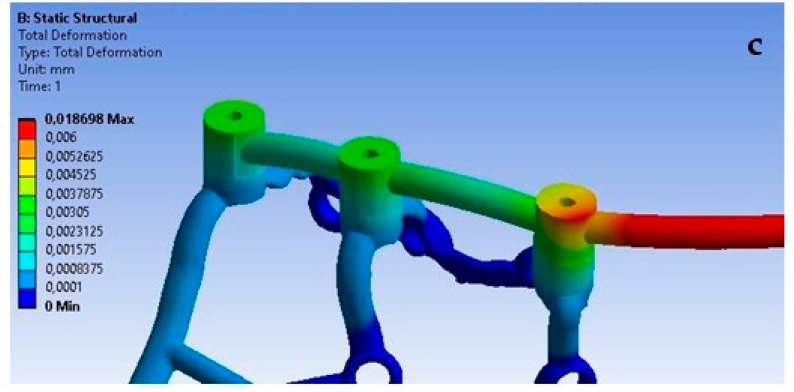
Displacement state for the arch under a load of 800 N at angles of (**a**) 30 degrees, (**b**) 60 degrees, and (**c**) 90 degrees.

Comparing both models, it is evident that with variable loads between 100 N and 800 N at a 90-degree angle, the single implant shows greater displacements on the internal side of the multi-units, which are on average 35% higher. For the arch, these displacements are larger on the external side, averaging 24% higher (Figure 18). For a load of 800 N with varying angles between 90 and 30 degrees, in the single implant, greater displacements occur on the internal side of the multi-units, averaging 46% higher. In the case of the arch, larger displacements appear on the internal side for angle variants of 60 and 30 degrees, averaging 39% higher. In contrast, for the 90-degree angle, they are 26% higher on the external side (Figure 19).

### 3.2. Shape Deformation

In Table 4, the deformation results of selected elements of a single implant (multi−units and mounting holes) and the arch (additionally the beam) are presented for a variable load between 100 N and 800 N at an angle of 90 degrees.

In Table 5, the deformation results of selected elements of the studied models (multi-units, mounting holes, and beam) are compiled for a load of 800 N with variable angles of 90, 60, and 30 degrees.

**Figure 20:**
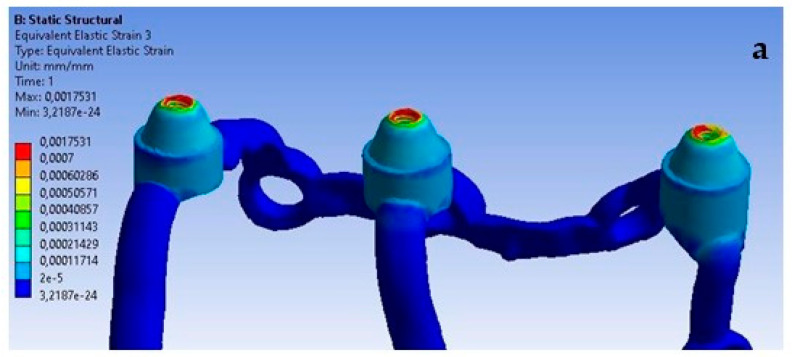

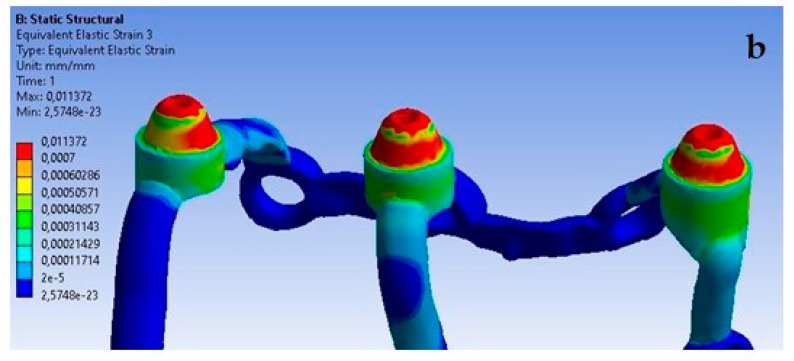
The state of strain for a single implant under forces of (**a**) 100 N and(**b**) 800 N.

The obtained results show that increasing the load from 100 N to 800 N at an angle of 90 degrees causes an increase in deformations throughout the entire single implant (Figure 20). The results on both sides vary in the range from 2.9 × 10^−5^ to 6.3 × 10^−4^ and they were permanent.

**Figure 21:**
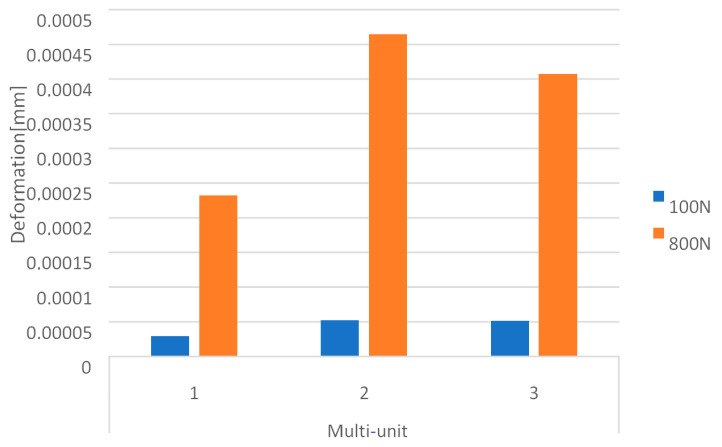
Deformations on the external side of multi-units for a single implant.

On the external side, the largest deformations occur in the second multi-unit for both load variants (Figure 21). As a result of increasing the force to 800 N, it was observed that the average deformation in all multi-units increased by 731%.

**Figure 22:**
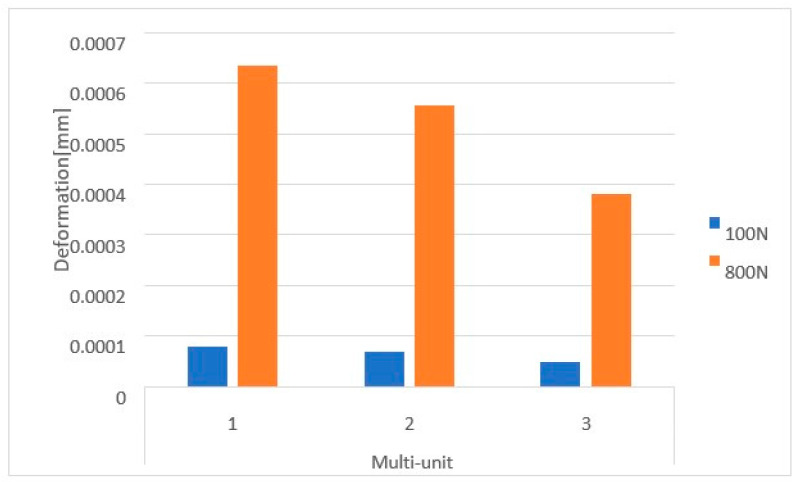
Deformations on the internal side of multi-units for a single implant.

On the internal side, the largest deformations were recorded in the first multi-unit for both load variants (Figure 22). An increase in load caused the average deformations in all multi-units to increase by 701%. On the internal side, there is also a mounting hole with the largest recorded deformations, which increased from 1 × 10^−5^ to 7.8 × 10^−5^.

**Figure 23:**
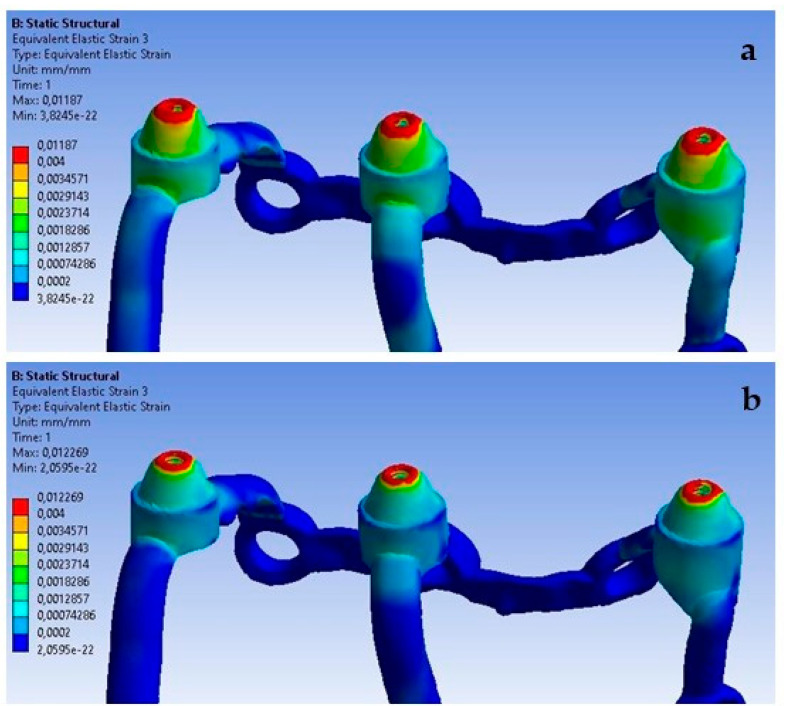

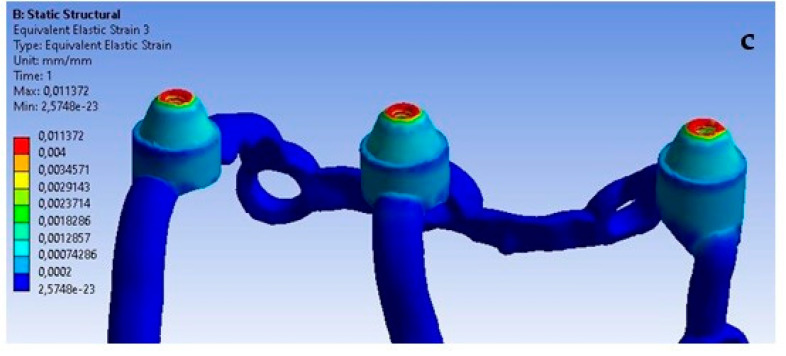
The state of strain for a single implant under a force of 800 N at angles of (**a**) 30 degrees, (**b**) 60 degrees, and (**c**) 90 degrees.

Changing the angle between 90 and 30 degrees affects the deformation results throughout the implant. They increase with its gradual reduction (Figure 23). On both sides of the multi-units, the values vary in the range from 2.3 × 10^−4^ to 3.8 × 10^−3^. This is larger compared to the variable load at an angle of 90 degrees.

### 3.3. Stresses

Table 6 presents the stress results for individual elements of a single implant (multi-units and mounting holes) and for the arch (additionally the bar) for a variable load in the range of 100–800 N at an angle of 90 degrees.

In Table 7, the stress results of the same elements are compiled for a load of 800 N with variable angles of 90, 60, and 30 degrees.

**Figure 24:**
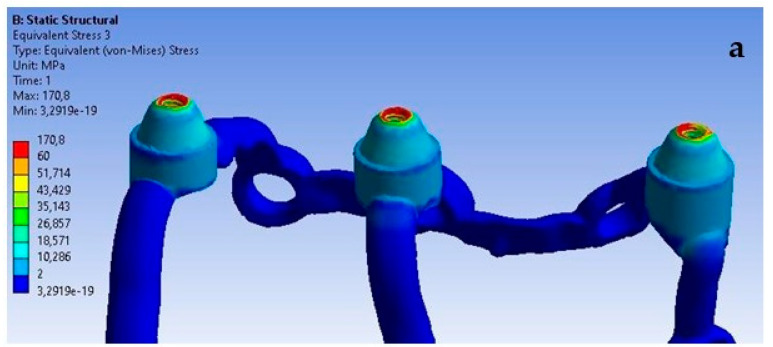

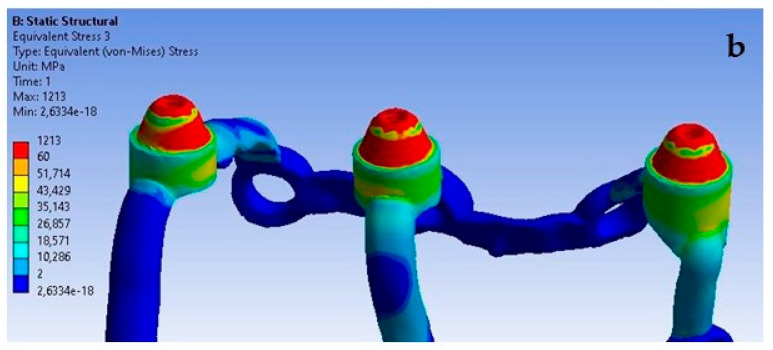
Stress state for a single implant under the forces of (**a**) 100 N and (**b**) 800 N.

For the last analyzed parameter, one can notice a similarity compared to displacements and deformations, that an increase in load simultaneously causes an increase in stresses throughout the entire single implant (Figure 24). In Figure 24, the stress values indicate a potential for microstructural damage or plastic deformation, which could impact the long-term integrity of the implant. This observation is critical for our ongoing research and development of these implants.

**Figure 25:**
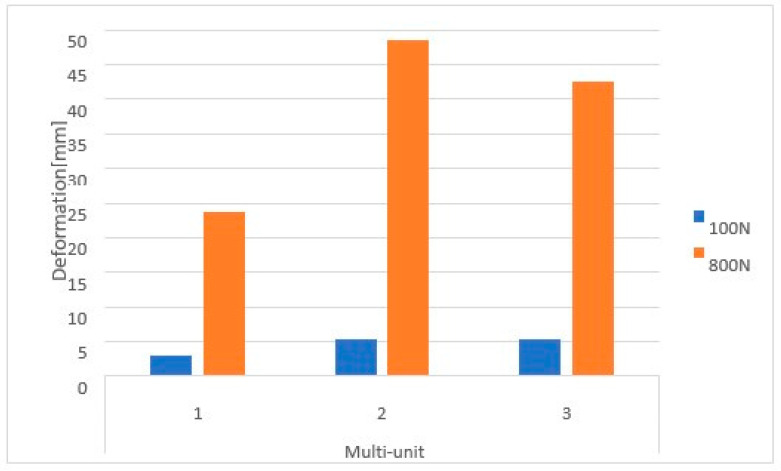
Stresses on the external side of the multi-units for a single implant.

On the external side, the highest stresses occur in the second multi-unit for both load variants (Figure 25). The increase in force caused the average stress in all multi-units to rise by 740%. The values range from 3 MPa to 48.6 MPa.

**Figure 26:**
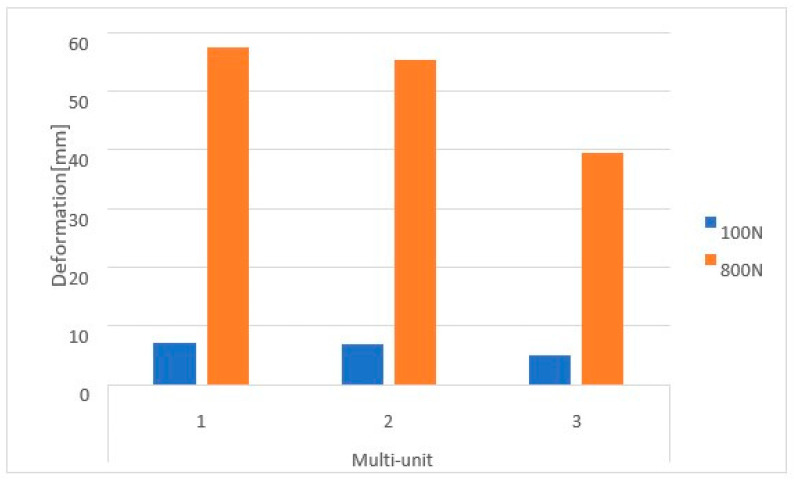
Stresses on the internal side of the multi-units for a single implant. Deformation was measured with 2 different forces—100 N and 800 N.

On the internal side, the first multi-unit is characterized by the highest stresses for both loads (Figure 26), which range from 5 MPa to 57.5 MPa. An increase of 701% in stresses was noted in all multi-units. On the internal side, there is a mounting hole with the highest stresses, which increased from 1 MPa to 7.8 MPa.

**Figure 27:**
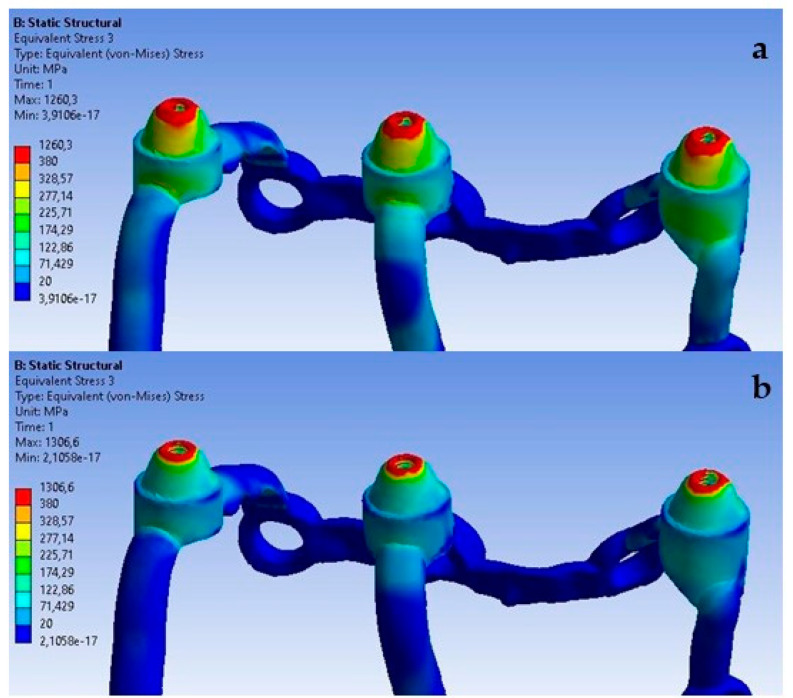

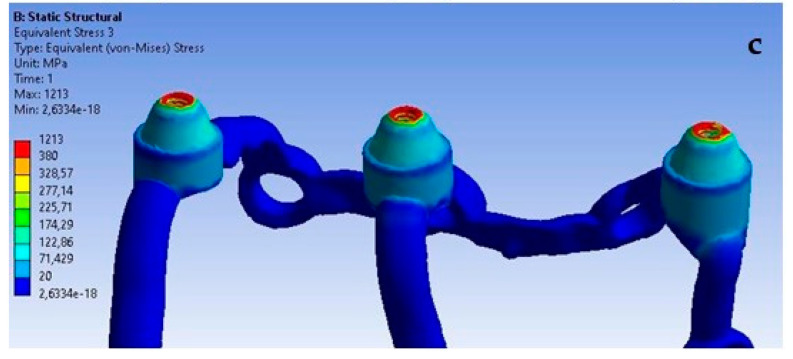
Stress state for a single implant under a load of 800 N at angles of (**a**) 30 degrees, (**b**) 60 degrees, and (**c**) 90 degrees.

For a load of 800 N with a variable angle, the results show that the lowest stresses occur for the 90-degree angle variant and increase linearly as it decreases to 30 degrees throughout the entire implant (Figure 27).

**Figure 28:**
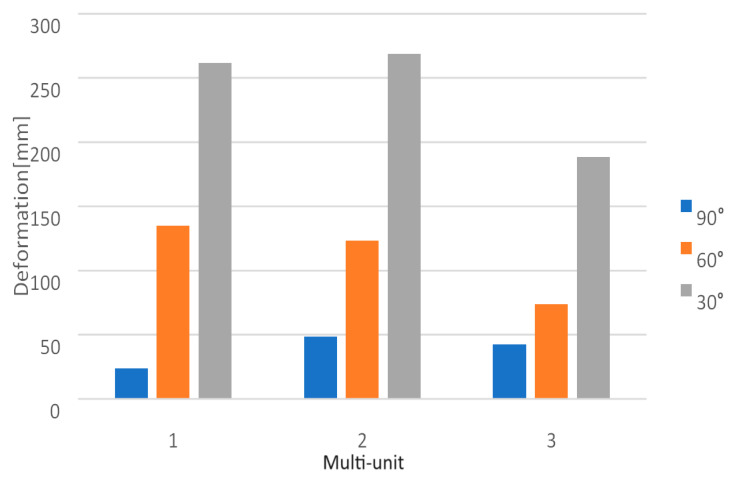
Stresses on the external side of the multi-units for a single implant. Deformation was measured with 3 different angles—30°, 60°, 90°.

On the external side, the highest stresses occur in the second multi-unit for angle variants of 90 and 30 degrees. For the 60-degree case, they occur in the first multi-unit (Figure 28). Stresses in the multi-units range from 23.7 MPa to 268.5 MPa. Changing the angle of load interaction from 90 to 30 degrees caused the average stress in all multi-units to increase by 526%.

**Figure 29:**
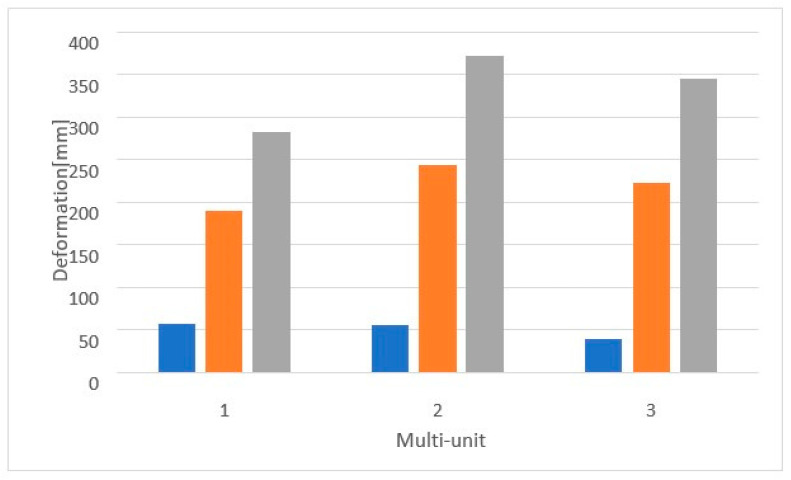
Stresses on the internal side of the multi-units for a single implant.

On the internal side, the highest stresses occur in the second multi-unit for angle variants of 60 and 30 degrees, while in the first they occur for the 90-degree angle (Figure 29). An increase of 556% in stresses was observed in all multi-units. Stresses in the multi-units range from 39.5 MPa to 371.7 MPa, whereas in the mounting hole with the highest stresses, they increase from 7.8 MPa to 57 MPa.

### 3.4. Analysis Results in the Absence of Bone Support for the Implant

The next step was to conduct a study for a single implant under a load of 800 N at a 30-degree angle from the vertical axis and to analyze the obtained results of displacements, deformations, and stresses. The analysis was conducted taking into account the fixation of the surface under the multi-units, that is, in the situation when the implant will be supported by bone, and without considering this surface, that is, in a situation where there will be a free space between the implant and the bone. In Table 8, the results of displacements on both sides of the multi-units and in the mounting holes with the highest recorded values for two variants of fixation were compiled.

In Table 9, the results of deformations on both sides of the multi-units and the mounting holes with the highest values are presented.

**Figure 30:**
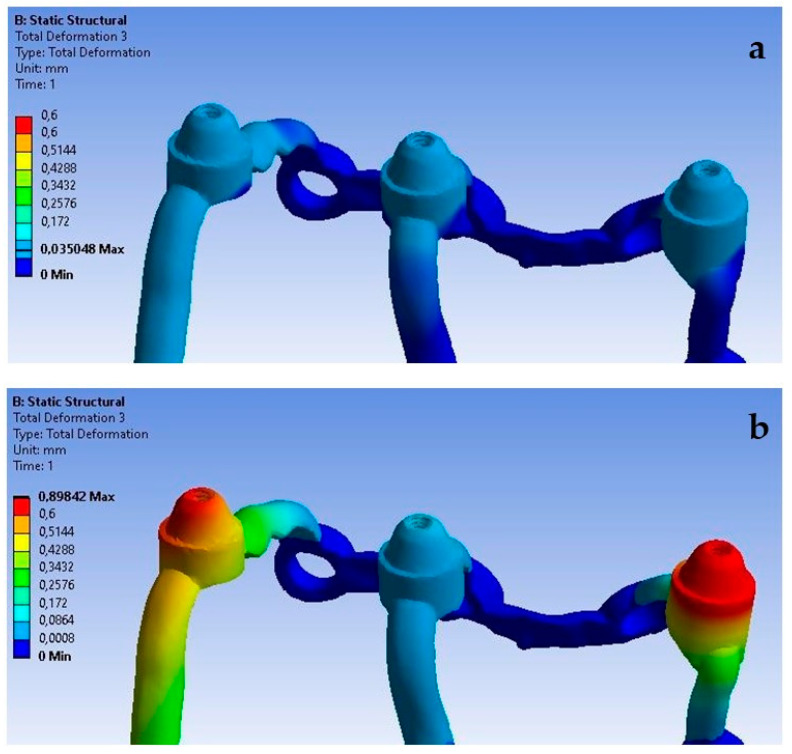
Displacements: (**a**) with multi-unit fixation, (**b**) without fixation.

The lack of support under the multi-units, which causes a free space between the implant and the bone, has the most significant effect on the displacement results. Ensuring the proper seating of the implant on the bone results in displacements of very small values in the range of 8.9 × 10^−4^ mm to 3.5 × 10^−3^ mm, while its absence contributes to implant displacements of even up to 0.5 mm. It can be noticed that the lack of support contributes to an uneven distribution of displacements, and they are larger at the first and third multi-units (Figure 30). The impact of the lack of fixation is also noticeable in the mounting holes. The largest displacements were recorded in the ninth hole, which is on the inner side of the first multi-unit.

**Figure 31:**
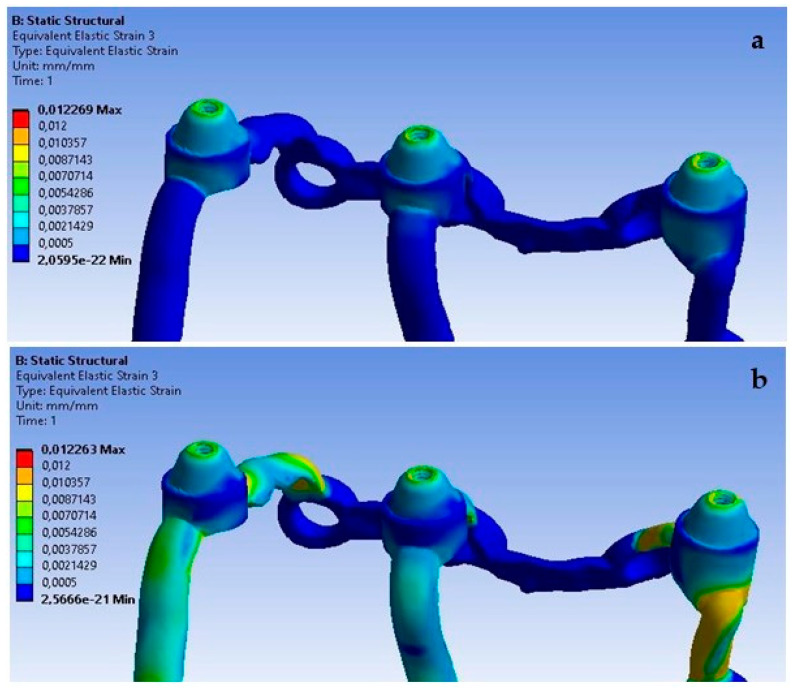
Deformations: (**a**) with fixation under multi-units, (**b**) without fixation.

An increase in deformations in a single implant is also noticeable but on a smaller scale compared to the previously described displacements (Figure 31). The values in the multi-units mainly change in the range from 10^−3^ to 10^−2^; however, a decrease in deformation values can be observed as a result of the lack of support on the outer side of the second multi-unit. The location of the greatest deformations among the holes has also changed, which were recorded in the ninth hole, similarly as in the case of displacements.

The red zones in the figures represent areas of maximum stress or strain within the analyzed structure, as determined by our Finite Element Analysis. These areas are subjected to the highest mechanical loads and are therefore most prone to material fatigue, deformation, or failure. The stress levels in these areas may exceed the material’s yield strength or fatigue limit, which could lead to irreversible deformation or fracture over time, particularly under cyclic loading conditions. These zones are of particular concern as they could be indicative of areas where failure is most likely to occur. This could have significant implications for the longevity and reliability of the implant. The insights provided by the analysis of the red zones are invaluable in guiding future improvements and ensuring the highest standards of safety and efficacy in our designs.

## 4. Discussion

### 4.1. FEA in Subperiosteal Implants

FEM analysis is a powerful simulation tool that allows you to evaluate structural behavior and stresses in a variety of situations [19,20]. However, like any modeling and simulation tool, there are some limitations that are important to consider when using FEA to assess stress variations. Among them, we find the accuracy of the input data. In fact, the accuracy of FEA results depends on the quality of the input data used, such as loading conditions and material properties [21].

The conducted numerical analysis using the Finite Element Analysis allowed for showing the state of displacements, deformations, and stresses in the studied implants, which serve to assess the risk from a biomechanical point of view. Such studies have a significant advantage over clinical trials, as patients are not subjected to tests, for example, in terms of using a new material. Thanks to the mathematical model, it is possible to properly fit the implant in terms of shape and size. Bite forces in the oral cavity depend on individual anatomical characteristics of the jaw and mandible, but in healthy individuals for molar teeth, they are 260 N for women and 360 N for men. The load of 100 N adopted in the studies is one of the most commonly accepted values for testing all kinds of dental implants, while a load of 800 N should be considered especially in patients with dysfunctions of the masticatory organ, in which bite forces significantly differ from the values occurring in a healthy person. Additionally, the conditions of loads with changing angles of 90, 60, and 30 degrees were adopted for the studies. Forces acting vertically, i.e., along the long axis of the teeth at a 90-degree angle, cause direct contact of opposing teeth, providing uniform and spatial support. From a mechanical point of view, this is a favorable situation, as the forces generated during chewing cause symmetrical muscle work.

Daily functioning or improper bite conditions also lead to the occurrence of forces at various angles, as a result of which stretching stresses considered destructive arise. This is reflected in the studies on subperiosteal implants, which showed that the smallest values of displacements, deformations, and stresses were obtained for vertical loads at a 90-degree angle. The effect of loads at 60 and 30 degrees increased the obtained results. Although the observed changes were of very small values from 1.6 × 10^−4^ mm to 6 × 10^−3^ mm for displacements and from 1.2 × 10^−4^ to 5.5 × 10^−3^ for deformations, it can be seen that between 90 and 30 degrees, the results increased by over 500%. Similarly, the stress results for both a single implant and an arch showed that the stresses in the multi-units reached values of 40–50 MPa in the case of a vertical force at a 90-degree angle and grew to nearly 500 MPa as a result of the action of non-axial forces.

### 4.2. Evolution of Subperiosteal Implants

The longevity of traditional subperiosteal implants has been substantiated by various studies [22,23,24]. However, numerous reviews have also highlighted issues including infections, both immediate and delayed exposure of the implant, bone loss, formation of fistulas, and implant looseness. These problems have led to significant distress for patients and a high rate of implant failure [22,23,24,25,26,27]. Endosteal implants addressed many of the issues encountered with earlier subperiosteal implant designs, providing better long-term outcomes and causing less patient discomfort [25,26,27,28].With the ability to be produced in bulk, coupled with their straightforward insertion and removal process in case they fail, endosteal implants emerged as the preferred choice for treatment starting in the 1980s.

Nonetheless, endosseous implants are not a universal solution for all clinical conditions. Successful placement of endosseous implants requires a certain level of bone density and volume [29]. In the absence of these conditions, there is a risk that the implant could fail, harm critical anatomical structures, or the installation may be unfeasible [30].While smaller and shorter implants offer an alternative, they are not suitable in cases where there is a considerable loss of bone mass in both the vertical and horizontal dimensions [31].

If the available bone volume is too low for implant placement, there are multiple regenerative methods available to increase the size of the alveolar ridge vertically and horizontally. These methods, though, are only viable when there is enough native bone to sustain the grafts. Autologous bone, which can be collected from different anatomical locations, can be used for onlay grafts. Many practitioners frequently employ this method, and it is deemed by several experts to be the benchmark for regenerative procedures [32,33,34].

The primary limitation of using free grafts is that their harvest inevitably cuts off the microcirculation, which hinders the re-establishment of blood supply to the graft. For a graft to survive and osteogenesis to occur, revascularization is essential. This process takes time, and during this period, the viability of osteocytes can be significantly reduced [35]. As a result, necrotic bone spots can develop, leading to graft resorption that is both undesirable and unpredictable. The extent of this resorption largely depends on the source of the bone graft. Studies have shown that mandibular block grafts used for augmenting the maxillary ridge experience resorption rates ranging from 5 to 28% [34,36,37]. Grafts from the iliac crest are reported to lose an average of 50% of their volume within six months after being placed in an atrophied maxilla [38].Research by Fourcade et al. (2019) showed an average resorption rate of 25% for both calvarial (parietal) and ramus bone grafts used in pre-implant augmentation of maxillary alveolar ridges [39].

To counteract the issue of high resorption rates, guided bone regeneration (GBR) is often used alongside bone grafting. This method utilizes bone substitutes and barriers to isolate the graft from non-osteogenic cells, which facilitates the proliferation of osteoblasts and prevents the invasion of connective and epithelial tissues. This technique typically results in decreased resorption and increased bone volume post-ridge augmentation [40,41].

Despite the variability in bone graft resorption rates, the overall success rates for implants placed in grafted bone remain high. Aghaloo et al. (2016) reviewed the implant outcomes in fully edentulous maxillae that underwent bone grafting [33]; they observed 2446 implants over a period of 1 to 12 years, noting survival rates from 73.3% to 100%. When GBR techniques were applied, survival rates ranged from 96.1% to 100%. Furthermore, Motamedian et al. (2016) documented success rates of 72.8% to 100% for 2647 implants placed in the atrophied maxilla with onlay grafts made from autologous bone [42].

Beyond onlay grafting and guided bone regeneration, the elevation of the maxillary sinus floor is another widely utilized method for increasing bone volume. Nevertheless, the success of this procedure is highly dependent on the skill with which it is executed. One of the main causes of failure during this procedure is the intraoperative tearing of the Schneiderian membrane. Membrane perforation occurs relatively often during surgery, with studies citing an incidence rate that varies widely, from 3.6% to 41.8% [43].When the membrane is torn, it is unable to serve its critical role in containing the graft, which is vital for bone regeneration [44].

Maxillary sinus floor augmentation typically confines the extent of maxillary reconstruction to the area of the premolars. In cases where the maxilla has significantly atrophied, severe ridge degradation often also occurs in the premaxillary area. This part of the ridge may not be suitable for augmentation. Despite these challenges, various research studies have reported high implant success rates, exceeding 90%, with observation periods ranging from one to eleven years [34,35,36,37,38,39,40,41,42,43,44,45].

The development of 3D printing technology for titanium and advancements in 3D planning software have renewed interest in the subperiosteal implant design. Cone Beam Computed Tomography (CBCT) can accurately depict a patient’s remaining bone volume, and the data from CBCT scans are utilized to create a precise virtual model of the bone using reconstruction software. This advancement significantly improves the custom fit of subperiosteal implants for individual patients.

Titanium (Ti) and its alloys are renowned for their biocompatibility, making them the materials of choice for various medical and dental applications. This is a stark contrast to the Vitallium alloy made of cobalt, chromium, and molybdenum, which was used in earlier subperiosteal implants made with the lost-wax technique and lacked the ability to integrate with soft tissue or bone. In the cranio-maxillofacial skeleton, there is minimal bone loss observed beneath titanium alloy osteosynthesis plates that are rigidly fixed [43]. Furthermore, in both trauma and orthognathic surgeries, over a quarter of the osteosynthesis materials become covered by bone over time [46,47].

The phenomenon of alveolar ridge resorption in patients without teeth has been extensively recorded in the literature [48,49]. The degree of bone loss varies among individuals, and significant differences in the rate of resorption at different times and locations within the same patient have been observed [31].

Initially, a rapid phase of bone loss is commonly seen within the first three months following tooth extraction. This phase is succeeded by a slower, yet persistent, resorption that continues over the individual’s lifetime [44]. Koodaryan and Hafezeqoran’s systematic review and meta-analysis in 2016 highlighted that the average early bone loss around maxillary and mandibular implants is approximately 1.5 mm in the first year after the final prosthesis is placed [50]. Subsequently, an annual bone loss rate of about 0.2 mm is observed. It is expected that the Maxilla/Mandible Individual Implants (MaI Implants^®^) would follow a similar pattern of bone loss after the first year of functioning. Clinical data indicate that patients with prostheses supported by AMSJI experienced an average alveolar ridge resorption of 0.33 mm (with a standard deviation of 0.76 mm) and 0.08 mm (with a standard deviation of 0.33 mm) at the wings and basal frame on the underlying zygomatic-maxillary bone one year after loading [51].

### 4.3. Segmentation Process of MaI Implants^®^

Several obstacles complicate the segmentation of CBCT data for the MaI Implants^®^, including the following: (1) compromised bone quality in some patients, impeding clear 3D visualization of the bone structure; (2) the titanium alloy used in MaI Implants^®^, which leads to beam-hardening artifacts; (3) the adjacent soft tissues; (4) the inadequate contrast resolution in many CBCT scans; and (5) a scarcity of underlying bone in certain instances. These issues contribute to the semi-automated segmentation technique’s susceptibility to human error during measurement. Moreover, the technique of surface-based superimposition, which relies solely on the outermost layer of the 3D structure for alignment, demands a high-quality surface to ensure precise superimposition [52].

### 4.4. Clinical Implication and Future Research

The analyzed models assumed proper implantation through fixing holes and settlement on the bone tissue of implants, which should provide proper stabilization in the oral cavity for future prosthetic supplements. However, a situation may occur unnoticed by the doctor where the implanted subperiosteal implant will not fully settle on the bone and there will be a small free space between the anatomical support site and the implant surface. This is shown by the results obtained during the analysis of the load of 800 N at an angle of 30 degrees from the vertical axis, taking into account the fixation of the implant under the multi-units and without fixation.

In summary, while no synthetic system is flawless within the human organism, the MaI Implants^®^ (Integra Implants, Lodz, Poland) stand out as a noteworthy option for addressing significant maxillary atrophy, surpassing extensive bone grafts and zygomatic implants. This is primarily because it enables immediate restoration of chewing function through a single surgical procedure, which often only necessitates local anesthesia. The effectiveness of the MaI Implants^®^ has been demonstrated and it holds considerable potential. However, their long-term efficiency is yet to be fully established and requires further validation through extended prospective studies or observational research. A forthcoming modification under consideration, which involves situating all connecting arms beneath the palatal gingival or the usage of a template that enables positioning the multi-units deeper, presents a new aspect for evaluation. Ensuring stable attachment of the epithelial junction to the titanium posts will pose a challenge, making additional research essential to enhance the MaI Implants’^®^ long-term success.

Time-varying masticatory loads may provide more reliable results as they can cause more criticality than static loads. The load was applied in a concentrated way on the anchor points, but the insertion of the bridge in Co-Cr and Ti could create a different stress distribution due to the variation in the mechanical properties of the two materials. These aspects need to be further investigated.

## 5. Conclusions

The lack of proper stabilization of the implant has the greatest impact on the results of displacements, which change in the range from 8.9 × 10^−4^ mm to even 0.5 mm. Because implant treatment is a multi-year process, such displacements are significant for the later positioning of the implant compared to the initial conditions.

## Figures and Tables

**Table 1:** Compilation of materials, load values, and angles used in the literature [10,11,12,13,14,15,16,17].

Authors	Material	Force [N]	Angle [°]
Bahrami et al.	Ti-6Al-4V	200	90
Burlibasa et al.	Ti-6Al-4V	50	90, 60, 45, 30, 0
Gultekin et al.	Ti-6Al-4V	100, 150	90
Kayumi et al.	-	40, 100, 200, 400, 800	90
Liu et al.	Ti-6Al-4V	100	90
Materac et al.	Ti-6Al-4V	100, 200, 500	90
Mommaerts	Ti-6Al-4V	100	90
PN-EN ISO 14801	-	800	30

**Table 2:** Compilation of displacement results for varying force values.

Element	Implant	Element	Side	Single Implant (mm)		Arch (mm)	
				100 N	800 N	100 N	800 N
Multi-unit	Left	1	outer	3.50 × 10^−5^	2.89 × 10^−4^	5.40 × 10^−5^	4.75 × 10^−4^
			inner	1.30 × 10^−4^	1.04 × 10^−5^	8.81 × 10^−5^	7.04 × 10^−4^
		2	outer	7.89 × 10^−5^	6.45 × 10^−4^	6.76 × 10^−5^	5.42 × 10^−4^
			inner	7.55 × 10^−5^	6.05 × 10^−4^	6.99 × 10^−5^	5.61 × 10^−4^
		3	outer	7.78 × 10^−5^	6.24 × 10^−4^	9.65 × 10^−5^	7.78 × 10^−4^
			inner	5.48 × 10^−5^	4.39 × 10^−4^	2.09 × 10^−5^	1.64 × 10^−4^
	Right	3	outer	-	-	9.91 × 10^−5^	7.94 × 10^−4^
			inner	-	-	2.22 × 10^−5^	1.70 × 10^−4^
		2	outer	-	-	8.07 × 10^−5^	6.41 × 10^−4^
			inner	-	-	6.85 × 10^−5^	5.46 × 10^−4^
		1	outer	-	-	5.65 × 10^−5^	4.50 × 10^−4^
			inner	-	-	9.07 × 10^−5^	7.28 × 10^−4^
Mounting holes	Left	8	-	2.76 × 10^−6^	2.38 × 10^−5^	5.69 × 10^−6^	4.35 × 10^−5^
	Right	8	-	-	-	2.52 × 10^−6^	1.41 × 10^−5^
Bar	Left	near 3	-	-	-	6.89 × 10^−4^	5.48 × 10^−3^
	-	middle	-	-	-	2.27 × 10^−3^	1.82 × 10^−2^
	Left	near 3	-	-	-	6.77 × 10^−4^	5.41 × 10^−3^

**Table 3:** Compilation of displacement results for varying angle values.

Element	Implant	Position	Side	Single Implant (mm)			Arch (mm)		
				90°	60°	30°	90°	60°	30°
Multi-unit	Left	1	outer	2.89 × 10^−4^	2.43 × 10^−3^	4.41 × 10^−3^	4.75 × 10^−4^	2.74 × 10^−3^	5.10 × 10^−3^
			inner	1.04 × 10^−3^	3.47 × 10^−3^	5.01 × 10^−3^	7.04 × 10^−4^	3.24 × 10^−3^	4.94 × 10^−3^
		2	outer	6.45 × 10^−4^	1.82 × 10^−3^	3.79 × 10^−3^	5.42 × 10^−4^	2.48 × 10^−3^	4.96 × 10^−3^
			inner	6.05 × 10^−4^	2.81 × 10^−3^	4.25 × 10^−3^	5.61 × 10^−4^	3.62 × 10^−3^	5.71 ×10^−3^
		3	outer	6.24 × 10^−4^	8.88 × 10^−4^	2.33 × 10^−3^	7.78 × 10^−4^	8.02 × 10^−4^	2.19 × 10^−3^
			inner	4.39 × 10^−4^	2.66 × 10^−3^	4.32 × 10^−3^	1.64 × 10^−4^	2.49 × 10^−3^	4.41 × 10^−3^
	Right	3	outer	-	-	-	7.94 × 10^−4^	8.58 × 10^−4^	1.98 × 10^−3^
			inner	-	-	-	1.70 × 10^−4^	2.77 × 10^−3^	4.87 × 10^−3^
		2	outer	-	-	-	6.41 × 10^−4^	3.02 × 10^−3^	5.96 × 10^−3^
			inner	-	-	-	5.46 × 10^−4^	3.62 × 10^−3^	5.73 × 10^−3^
		1	outer	-	-	-	4.50 × 10^−4^	2.75 × 10^−3^	5.12 ×10^−3^
			inner	-	-	-	7.28 × 10^−4^	3.34 × 10^−3^	5.03 × 10^−3^
Mounting holes	Left	8	-	2.38 × 10^−5^	1.15 × 10^−4^	1.74 × 10^−4^	4.35 × 10^−5^	2.77 × 10^−4^	4.55 × 10^−4^
	Right	8	-	-	-	-	1.41 × 10^−5^	1.45 × 10^−4^	2.40 × 10^−4^
Bar	Left	near 3	-	-	-	-	5.48 × 10^−3^	2.24 × 10^−2^	3.94 × 10^−2^
	-	middle	-	-	-	-	1.82 × 10^−2^	1.00 × 10^−1^	1.60 × 10^−1^
	Left	near 3	-	-	-	-	5.41 × 10^−3^	2.28 × 10^−2^	3.95 × 10^−2^

**Table 4:** Compilation of deformation results for a variable force value.

Element	Implant	Position	Side	Single Implant (%)		Arch (%)	
				100 N	800 N	100 N	800 N
Multi-unit	Left	1	outer	2.92 × 10^−5^	2.32 × 10^−4^	5.39 × 10^−5^	4.30 × 10^−4^
			inner	7.90 × 10^−5^	6.34 × 10^−4^	7.29 × 10^−5^	5.88 × 10^−4^
		2	outer	5.24 × 10^−5^	4.65 × 10^−4^	6.34 × 10^−5^	5.06 × 10^−4^
			inner	6.84 × 10^−5^	5.55 × 10^−4^	6.12 × 10^−5^	4.89 × 10^−4^
		3	outer	5.13 × 10^−5^	4.07 × 10^−4^	8.43 × 10^−5^	6.69 × 10^−4^
			inner	4.85 × 10^−5^	3.81 × 10^−4^	1.60 × 10^−5^	1.23 × 10^−4^
	Right	3	outer	-	-	7.26 × 10^−5^	5.80 × 10^−4^
			inner	-	-	1.64 × 10^−5^	1.25 × 10^−4^
		2	outer	-	-	4.82 × 10^−5^	3.67 × 10^−4^
			inner	-	-	6.53 × 10^−5^	5.20 × 10^−4^
		1	outer	-	-	5.02 × 10^−5^	4.05 × 10^−4^
			inner	-	-	7.09 × 10^−5^	5.73 × 10^−4^
Mounting holes	Left	8	-	1.00 × 10^−5^	7.77 × 10^−5^	1.30 × 10^−5^	9.66 × 10^−5^
	Right	8	-	-	-	6.54 × 10^−5^	8.46 × 10^−5^
Bar	Left	Near 3	-	-	-	6.57 × 10^−5^	5.46 × 10^−4^
	-	Middle	-	-	-	2.76 × 10^−7^	1.56 × 10^−6^
	Right	Near 3	-	-	-	6.97 × 10^−5^	5.60 × 10^−4^

**Table 5:** Compilation of deformation results for a variable angle value.

Element	Implant	Position	Side	Single Implant (%)			Arch (%)		
				90°	60°	30°	90°	60°	30°
Multi-unit	Left	1	outer	2.32 ×10^−4^	1.38 ×10^−3^	2.65 ×10^−3^	4.30 ×10^−4^	2.24 × 10^−3^	4.33 × 10^−3^
			inner	6.34 × 10^−4^	2.22 × 10^−3^	3.26 × 10^−3^	5.88 × 10^−4^	2.89 × 10^−3^	4.55 × 10^−3^
		2	outer	4.65 × 10^−4^	1.18 × 10^−3^	2.59 × 10^−3^	5.06 × 10^−4^	2.29 × 10^−3^	4.49 × 10^−3^
			inner	5.55 × 10^−4^	2.46 × 10^−3^	3.78 × 10^−3^	4.89 × 10^−4^	3.18 × 10^−3^	4.92 × 10^−3^
		3	outer	4.07 × 10^−4^	7.17 × 10^−4^	1.83 × 10^−3^	6.69 × 10^−4^	8.37 × 10^−4^	2.17 × 10^−3^
			inner	3.81 × 10^−4^	2.19 × 10^−3^	3.39 × 10^−3^	1.23 × 10^−4^	2.66 × 10^−3^	4.58 × 10^−3^
	Right	3	outer	-	-	-	5.80 × 10^−4^	6.55 × 10^−4^	1.76 × 10^−3^
			inner	-	-	-	1.25 × 10^−4^	2.56 × 10^−3^	4.39 × 10^−3^
		2	outer	-	-	-	3.67 × 10^−4^	1.82 × 10^−3^	3.66 × 10^−3^
			inner	-	-	-	5.20 × 10^−4^	3.94 × 10^−3^	5.51 × 10^−3^
		1	outer	-	-	-	4.05 × 10^−4^	2.22 × 10^−3^	4.27 × 10^−3^
			inner	-	-	-	5.73 × 10^−4^	2.89 × 10^−3^	4.40 × 10^−3^
Mounting holes	Left	8	-	7.77 × 10^−5^	3.76 × 10^−4^	5.71E−04	9.66 × 10^−5^	6.75 × 10^−4^	1.03 × 10^−3^
	Right	8	-	-	-	-	6.54 × 10^−5^	4.51 × 10^−4^	7.24 × 10^−4^
Bar	Left	near 3	-	-	-	-	5.46 × 10^−4^	8.10 × 10^−4^	1.13 × 10^−3^
	-	middle	-	-	-	-	1.56 × 10^−6^	5.96 × 10^−5^	1.39 × 10^−4^
	Right	near 3	-	-	-	-	5.60 × 10^−4^	8.80 × 10^−4^	1.22 × 10^−3^

**Table 6:** Compilation of stress results for a variable load value.

Element	Implant	Position	Side	Single Implant MPa		Arch (MPa)	
				100 N	800 N	100 N	800 N
Multi-unit	Left	1	outer	3.0	23.7	5.5	44.6
			inner	7.1	57.5	6.9	56.3
		2	outer	5.4	48.6	6.5	51.7
			inner	7.0	55.4	5.8	48.3
		3	outer	5.3	42.4	8.9	70.6
			inner	5.0	39.5	1.7	13.0
	Right	3	outer	-	-	7.7	60.9
			inner	-	-	1.7	13.0
		2	outer	-	-	5.0	37.9
			inner	-	-	6.7	54.7
		1	outer	-	-	5.2	41.4
			inner	-	-	6.8	55.9
Mounting holes	Left	8	-	1.0	7.8	1.3	9.7
	Right	8	-	-	-	0.7	4.9
Bar	Left	near 3	-	-	-	6.7	55.9
	-	middle	-	-	-	2.4 × 10^−2^	0.1
	Right	near 3	-	-	-	7.1	56.2

**Table 7:** Compilation of stress results for a variable angle value.

Implant	Position	Side	Single Implant, MPa				Arch, MPa	
			90°	60°	30°	90°	60°	30°
Left	1	Outer	23.7	134.7	261.5	44.6	227.5	440.5
		inner	57.5	189.4	283.5	56.3	274.8	416.5
	2	outer	48.6	123.0	268.5	51.7	233.8	462.6
		inner	55.4	243.9	371.7	48.3	303.0	481.0
	3	outer	42.4	73.4	188.2	70.6	88.5	228.5
		inner	39.5	223.5	344.9	13.0	277.5	481.0
Right	3	outer	-	-	-	60.9	68.7	176.6
		inner	-	-	-	13.0	269.0	460.0
	2	outer	-	-	-	37.9	181.0	370.6
		inner	-	-	-	54.7	371.0	567.0
	1	outer	-	-	-	41.4	227.3	436.7
		inner	-	-	-	55.9	270.0	417.0
Left	8	-	7.8	37.7	57.1	9.7	67.1	103.0
Right	8	-	-	-	-	4.9	40.0	70.2
Left	near 3	-	-	-	-	55.9	81.3	112.0
-	middle	-	-	-	-	0.1	5.1	15.0
Right	near 3	-	-	-	-	56.2	86.0	119.0

**Table 8:** Displacement results for a single implant.

Element	Position	Side	Anchorage under Multi-Units, mm	
			Yes	No
Multi-unit	1	outer	2.4 × 10^−3^	0.5
		inner	3.5 × 10^−3^	0.4
	2	outer	1.8 × 10^−3^	2.8 × 10^−2^
		inner	2.8 × 10^−3^	8.7 × 10^−2^
	3	outer	8.9 × 10^−4^	0.4
		inner	2.7 × 10^−3^	0.4
Mounting hole	8	-	1.1 × 10^−4^	-
	9	-	-	1.9 × 10^−3^

**Table 9:** Deformation results for a single implant.

Element	Position	Side	Anchorage under Multi-Units, %	
			Yes	No
Multi-unit	1	outer	1.4 × 10^−3^	4.1 × 10^−3^
		inner	2.2 × 10^−3^	1.1 × 10^−2^
	2	outer	1.2 × 10^−3^	5.9 × 10^−4^
		inner	2.5 × 10^−3^	1.1 × 10^−2^
	3	outer	7.2 × 10^−4^	1.1 × 10^−2^
		inner	2.2 × 10^−3^	1.1 × 10^−2^
Mounting hole	8	-	3.8 × 10^−4^	-
	9	-	-	6.1 × 10^−3^

The highest values are presented.

## Data Availability

Data are contained within the article.
